# Hybrid 2-Quinolone–1,2,3-triazole Compounds: Rational Design, In Silico Optimization, Synthesis, Characterization, and Antibacterial Evaluation

**DOI:** 10.3390/antibiotics14090877

**Published:** 2025-08-30

**Authors:** Ayoub El-Mrabet, Abderrahim Diane, Rachid Haloui, Hanae El Monfalouti, Ashwag S. Alanazi, Mohamed Hefnawy, Mohammed M. Alanazi, Youssef Kandri-Rodi, Souad Elkhattabi, Ahmed Mazzah, Amal Haoudi, Nada Kheira Sebbar

**Affiliations:** 1 Laboratory of Applied Organic Chemistry, Faculty of Sciences and Techniques, Sidi Mohamed Ben Abdellah University, Route d’Imouzzer, P.O. Box 2202, Fez 30000, Morocco; abderrahim.diane@usmba.ac.ma (A.D.); youssef.kandrirodi@usmba.ac.ma (Y.K.-R.); amal.haoudi@usmba.ac.ma (A.H.); 2 Laboratory of Engineering, Systems and Applications, National School of Applied Sciences, Sidi Mohamed Ben Abdellah-Fez University, P.O. Box 2202, Fez 30040, Morocco; rachid.haloui@usmba.ac.ma (R.H.); souad.elkhattabi@usmba.ac.ma (S.E.); 3 Laboratory of Plant Chemistry, Faculty of Sciences, Mohammed V University in Rabat, 4 Avenue 9 Ibn Battouta, P.O. Box 1014, Rabat 10000, Morocco; h.elmonfalouti@um5r.ac.ma; 4 Department of Pharmaceutical Sciences, College of Pharmacy, Princess Nourah bint Abdulrahman University, Riyadh 11671, Saudi Arabia; asalanzi@pnu.edu.sa; 5 Department of Pharmaceutical Chemistry, College of Pharmacy, King Saud University, Riyadh 11451, Saudi Arabia; mhefnawy@ksu.edu.sa (M.H.); mmalanazi@ksu.edu.sa (M.M.A.); 6 University of Lille, CNRS, UAR 3290, MSAP, Miniaturization for Synthesis, Analysis and Proteomics, 59000 Lille, France; ahmed.mazzah@univ-lille.fr; 7 Laboratory of Heterocyclic Organic Chemistry, Pharmacochemistry Competence Center, Faculty of Sciences, Mohammed V University in Rabat, 4 Avenue 9 Ibn Battouta, P.O. Box 1014, Rabat 10000, Morocco

**Keywords:** 1,2,3-triazole, 2-quinolone, antibacterial activity, antibiotic resistance, drug discovery, hybrid molecules, molecular docking, QSAR modeling

## Abstract

**Background/Objectives:** The rise in antibiotic resistance presents a serious and urgent global health challenge, emphasizing the need to develop new therapeutic compounds. This study focuses on the design and evaluation of a novel series of hybrid molecules that combine the 2-quinolone and 1,2,3-triazole pharmacophores, both recognized for their broad-spectrum antimicrobial properties. **Methods:** A library of 29 candidate molecules was first designed using in silico techniques, including QSAR modeling, ADMET prediction, molecular docking, and molecular dynamics simulations, to optimize antibacterial activity and drug-like properties. The most promising compounds were then synthesized and characterized by ^1^H and ^13^C NMR APT, mass spectrometry (MS), Fourier-transform infrared (FT-IR) spectroscopy, and UV-Vis spectroscopy. **Results:** Antibacterial evaluation revealed potent activity against both Gram-positive and Gram-negative bacterial strains, with minimum inhibitory concentration (MIC) values ranging from 0.019 to 1.25 mg/mL. **Conclusions:** These findings demonstrate the strong potential of 2-quinolone–triazole hybrids as effective antibacterial agents and provide a solid foundation for the development of next-generation antibiotics to combat the growing threat of bacterial resistance.

## 1. Introduction

The emergence of antibiotic resistance is a global problem and a serious threat [1,2]. Antimicrobial resistance is currently responsible for approximately 700,000 deaths per year, and based on current trends and speculation, this number is expected to rise to approximately 10 million in a few years [3]. These statistics underscore the urgent need to develop novel antibacterial agents that can combat both drug-susceptible and drug-resistant infections [4]. A primary focus of organic, medicinal, and heterocyclic chemistry is the synthesis and design of compounds to address therapeutic points in human health. Heterocyclic chemistry is a prominent branch of chemical sciences that has emerged as a significant area of research, driven by the search for solutions to address adverse conditions associated with human survival problems [5,6]. In this context, quinolone derivatives have reportedly received significant attention due to their remarkable medicinal properties [7]. These scaffolds constitute the structural components of many, if not all, natural products and are crucial in drug development due to their therapeutic efficacy [8,9]. Quinolones and their derivatives share specific characteristics, including a bicyclic aromatic nucleus with a nitrogen atom in the 1-position and a ketone functional group [10]. Quinolones and their derivatives are considered “privileged building blocks” for libraries of bioactive molecules, owing to their simplicity and adaptability in synthetic procedures. Due to their wide range, high availability, and safety profiles, they are highly favored platforms for developing chemical libraries with overwhelming bioactivity [11,12]. Moreover, quinolones are among the most widely prescribed drugs worldwide and are widely accepted due to their broad spectrum of action, high bioavailability, and high safety profile.

Modifications of the parent structure of nalidixic acid, for example, have resulted in a series of compounds with broad therapeutic utility. Extensive biological activity studies have been conducted on important quinolones, including norfloxacin, ciprofloxacin, ofloxacin, and their related derivatives [13,14]. Structure–Activity Relationship (SAR) studies have played a crucial role in providing the chemistry necessary to develop highly potent compounds for testing against various diseases. Quinolone derivatives are known to exhibit a wide range of biological activities, including antibacterial [11,14,15], antifungal [16], analgesic [17], anti-inflammatory [18], larvicidal [12], anticancer [19], antitubercular [20], insecticidal [21], antimalarial [22], anti-HIV [23], anti-hepatitis C virus [24], and anti-Alzheimer activities [25]. Quinolones, a class of antibiotics, are first-line treatments for acute urinary tract infections and bacterial diarrhea. Several analogs can be made with substituted patterns of the quinolone core skeleton, thereby exploring further lead development with better efficacy and pharmacokinetic properties [26]. This study aims to construct QSAR (Quantitative Structure-Activity Relationship) models using an in silico approach to describe and predict the antibacterial inhibitory activity of 2-oxo-1,2-dihydroquinoline derivatives, leveraging diverse molecular descriptors. Additionally, the work involves designing and synthesizing novel compounds based on the 2-quinolone core structure, followed by their characterization through spectroscopic methods (^1^H NMR, ^13^C NMR APT, MS, FT-IR, and UV-Vis) and assessment of their antibacterial efficacy. In silico evolution of ADMET properties, molecular docking, and molecular dynamics are employed to investigate the binding interactions of these quinolone derivatives with the active site of the LasR-OC12 HSL receptor, as obtained from the Protein Data Bank (PDB ID: 3IX3), providing insights into their mode of action. These computational analyses guide the rational design of structurally optimized molecules with enhanced biological potency, streamlining the synthesis process by prioritizing promising candidates before experimental validation.

## 2. Materials and Methods

### 2.1. Chemical Reactions

**Scheme 1:**
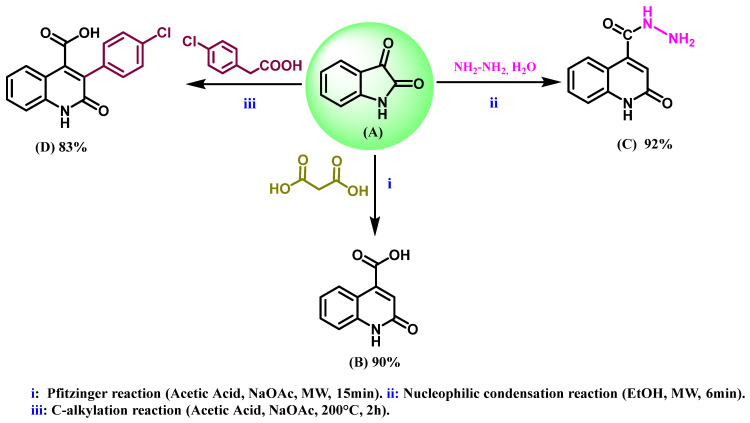
Synthesis methods for synthesizing 2-oxo-1,2-dihydroquinoline-4-carboxylic acid from isatin.

In our search for new bioactive compounds based on the quinoline scaffold derived from the isatin molecule [27], we identified four practical synthetic approaches used to produce quinolinic acid, each showing notable features. The Pfitzinger reaction [28], carried out under microwave irradiation with acetic acid and sodium acetate, enables rapid and efficient (90%) formation of the quinolinic acid backbone, demonstrating the benefits of a mild and time-saving method. The microwave-assisted reaction reported by El Ashry et al. [29], which involves treating isatin with hydrazine hydrate in water, yields quinoline-2,3-dione derivatives in excellent amounts. This eco-friendly method underscores the efficiency of microwave irradiation in accelerating heterocyclization under mild aqueous conditions, offering a valuable alternative to traditional heating methods. Finally, the method used by Tsung-Chih Chen [30], involving an α-halo acid in acetic acid at high temperature (200 °C), allows for the addition of chlorine atoms, which are essential in medicinal chemistry, with an 83% yield. Overall, these methods showcase the versatility and synthetic richness of the isatin core in accessing diverse and promising compounds for new drug development [31]. In our lab, we chose the Pfitzinger reaction due to its high efficiency and selectivity in forming new compounds. These compounds were then tested against various bacterial strains (Scheme 1).

### 2.2. Experimental Databases

**Scheme 2:**
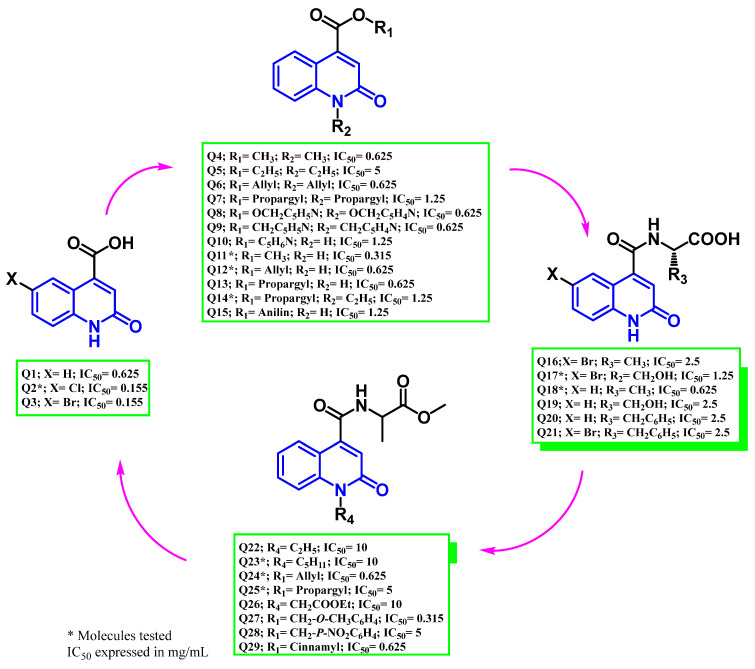
The quinolones compounds and their antibacterial activities.

A set of 29 compounds based on 2-oxo-1,2-dihydroquinoline carboxylic acid derivatives (Scheme 2), synthesized by Moussaoui et al. [15,32] and Fillali et al. [33,34,35], and tested for their antibacterial activity, served as the basis for the development of a QSAR model. The development method begins with drawing the structure of each compound in ACD/ChemSketch Freeware, version 2022.2 (Advanced Chemistry Development, Toronto, ON, Canada) and saving it in mol format. Then, using the RDKit package (version 2024.9.5), these 29 mol files were loaded into Python (version 3.11) for structural optimization and molecular descriptor computation.

The generated descriptors were structured as a 2D array, with each row representing a chemical and each column containing the value of a specific descriptor for that compound. This array was then aligned with a 1D vector holding the IC_50_ value of each drug [36].

After the data was prepared, it was manually divided into training and testing subsets, with the data from 20 compounds used to develop the QSAR model and that from the remaining 9 compounds used for model validation. This ensured that the training set included the entire range of IC_50_ values. A wrapper descriptor selection technique was used to reduce the dimensionality of the data. This technique iteratively evaluated the root mean squared error (RMSE) from 5-fold cross-validation of a multiple linear regression model (provided by scikit-learn v1.6.1) with various descriptor combinations. The tree-structured Parzen estimator provided by the hyperopt (v0.2.7) package was used to optimize the iterative selection process, to minimize RMSE.

Once the selection process is complete, the subset of descriptors used to develop the MLR model with the lowest RMSE is used to create a symbolic regression (SR) model. The goal of this step was to create a model that outperforms the MLR model. The root mean squared error and R-squared were used to evaluate the performance of the developed symbolic model [37]. This thorough evaluation provided more profound insights into the relationship between molecular descriptors and biological activity, contributing to the development of more accurate and reliable QSAR models for future applications [38,39].

### 2.3. Spectral Data Measurements

The NMR spectra of the synthesized derivatives were recorded on a Bruker Avance DPX300 spectrometer (Billerica, MA, USA). Deuterated chloroform (CDCl_3_) or DMSO-*d*_6_ was used as solvent, depending on the solubility of the compound. Tetramethylsilane (TMS) was used as the internal reference, and all chemical shifts (δ) are reported in parts per million (ppm) relative to TMS. ^1^H and ^13^C NMR spectra were recorded under standard conditions and Attached Proton Test (APT) experiments were performed to assign carbon multiplicities (C, CH, CH_2_, CH_3_). The interpretation was based on chemical shifts, signal patterns, integrals, and APT phase behavior. The singlet observed at δ = 7.26 ppm in the ^1^H NMR spectrum of compound 4a corresponds to the residual proton signal of the CDCl_3_ solvent and was not assigned to the compound.

All other solvents and reagents used were analytical grade. On silica gel plates (60 F_254_, Merck, Darmstadt, Germany), TLC was performed; compounds were purified using silica gel (230–400 mesh, Merck) [40]. Mass spectrometry was used, which included a 337 nm UV laser (Spectra-Physics, Santa Clara, CA, USA) and a 7 Tesla superconducting magnet (Bruker BioSpin, Billerica, MA, USA). DHB served as the matrix, formulated at 10 mg/mL in a 1:1 (*v*/*v*) combination of acetonitrile and water, including 0.1% trifluoroacetic acid. Samples were solubilized in methanol, and a 1 µL aliquot of the sample/matrix combination (1:1, *v*/*v*) was applied. Measurements were conducted in positive ion mode with internal calibration using standard references. The data were examined with the instrument’s included software (Data acquisition and analysis were carried out with flexAnalysis, version 3.4 (Bruker Daltonics, Bremen, Germany)) [40]. FT-IR spectra were obtained with a Vertex 70 spectrometer (Bruker, Shanghai, China) at a resolution of 4 cm^−1^ over 32 scans, spanning the 400–4000 cm^−1^ range [40]. UV-Vis measurements were conducted using a Bio-based BK-D580 double-beam spectrophotometer. Melting points were determined using a computerized melting point apparatus.

### 2.4. Synthesis of New Antibacterial Agents Based on the Quinolone Derivatives

#### 2.4.1. Synthesis of 2-Oxo-1,2-dihydroquinoline-4-carboxylic Acid (**1**)

A mixture of isatin (13 mmol), malonic acid (16 mmol), and sodium acetate (2 mmol) was dissolved in acetic acid as a reaction medium. The mixture was refluxed for 24 h. After cooling to room temperature, water was added to the reaction mixture, resulting in the formation of a precipitate **1**. The precipitate was collected by filtration [6].

#### 2.4.2. Esterification and *N*-Alkylation Reaction (Synthesis of Compounds **2** and **3**)

To a solution containing Compound **1** (10 mmol), 15 mL of methanol and 1 mL of sulfuric acid were added. The reaction mixtures were kept under stirring at reflux for 2 h. Pure compound **2** was obtained by precipitation in DMSO/H_2_O [41].

Methyl 2-oxo-1,2-dihydroquinoline-4-carboxylate (4 mmol) **3** was dissolved in 12 mL of dimethylformamide (DMF), followed by the addition of propargyl bromide (10 mmol), potassium carbonate (K_2_CO_3_, 20 mmol), and tetra-n-butylammonium bromide (TBAB, 0.5 mmol). The reaction mixture was stirred at room temperature for 6 h. Afterward, the resulting salts were filtered out, and the solvent was removed under reduced pressure. The remaining residue was then dissolved in dichloromethane [41].

#### 2.4.3. Synthesis of New Hybrid Molecules (**4a**–**4c**) Using 1,3-Dipolar Cycloaddition (Click Chemistry)

A solution of compound **3** (1 mmol) and alkyl azide (2 mmol) was prepared in a 1:2 (*v*/*v*) water/ethanol mixture at room temperature. Copper (II) sulfate pentahydrate (1 mmol) and sodium ascorbate (1 mmol) were then added. The reaction was stirred at room temperature before the solvent was evaporated under reduced pressure. The residue was extracted three times with a mixture of water and chloroform. The organic phase was recovered, dried over sodium sulfate, filtered and concentrated in vacuo. The crude product was purified by silica gel column chromatography using a mixture of ethyl acetate and hexane (3:1) as eluent. The three newly synthesized compounds were characterized by standard spectroscopic methods, including ^1^H NMR, ^13^C NMR, MS, FT-IR and UV-Vis spectroscopy.

*Methyl 1-((1-(4-Bromobenzyl)-1H-1,2,3-triazol-4-yl)methyl)-2-oxo-1,2-dihydroquinoline-4-carboxylate* (**4a**). **Yield (%) =** 78%; **mp:** 439–441 K; **NMR ^1^H (300 MHz, CDCl_3_): δ** (ppm) 8.2 (dd, *J* = 8.3, 1.5 Hz, 1H, CH_Ar_), 7.9 (d, *J* = 8.6 Hz, 1H, CH_Ar_), 7.6–7.5 (m, 2H, CH_Ar_), 7.4 (d, *J* = 8.3 Hz, 2H, CH_Ar_), 7.2 (d, *J* = 2.8 Hz, 1H, CH_Ar_), 7.1 (s, 1H, CH_Ethylenic_), 7.1 (d, *J* = 8.3 Hz, 2H, CH_Ar_), 5.6 (s, 2H, CH_2_), 5.3 (s, 2H, CH_2_), 3.9 (s, 3H, CH_3_). **^13^C NMR APT (75 MHz, CDCl_3_): δ** (ppm)165.85 (C=O_ester_), 161.3 (C=O_Amide_), 141.1 (Cq), 139.6 (Cq), 139.6 (Cq), 133.3 (CH_Ar_), 132.4 (CH_Ar_), 131.7 (CH_Ar_), 129.9 (CH_Ar_), 127.2 (CH_Ethylenic_), 123.8 (CH_Ar_), 123.1 (CH_Ar_), 117.6 (Cq), 115.6 (CH_Ar_), 53.7 (CH_2_), 53.0 (CH_2_), 38.7 (CH_3_). **MS:** 452.50677 [M + 1]+; **FT-IR (υ in cm^−1^):** 2961 (Aliphatic C–H), 1731 (C=O Ester), 1644 (C=O Ketone in quinolone), 1433 (Aromatic C=C stretching), 3125 (Aromatic C-H stretching), 1238 (C-N triazole bond); **UV–vis**: λ_max_ = 375 nm.
*Methyl 1-((1-(4-Methylbenzyl)-1H-1,2,3-triazol-4-yl)methyl)-2-oxo-1,2-dihydroquinoline-4-carboxylate* (**4b**). **Yield (%) =** 82%; **mp:** 488–490 K; **NMR ^1^H (300 MHz, CDCl_3_): δ** (ppm) 8.1–8.0 (m, 2H, CH_Ar_), 7.7 (d, *J* = 8.6 Hz, 1H, CH_Ar_), 7.6–7.6 (m, 1H, CH_Ar_), 7.3–7.2 (m, 1H, CH_Ar_), 7.1 (d, *J* = 10.4 Hz, 4H, CH_Ar_), 7.0 (s, 1H, CH_Ethylenic_), 5.5 (s, 2H, CH_2_), 5.4 (s, 2H, CH_2_), 3.9 (s, 3H, CH_3_), 2.2 (s, 3H, CH_3_). **^13^C NMR APT (75 MHz, CDCl_3_): δ** (ppm) 165.4 (C=O_ester_), 159.9 (C=O_Amide_), 142.6 (Cq), 139.2 (Cq), 137.5 (Cq), 132.9 (CH_Ar_), 131.4 (CH_Ar_), 129.2 (CH_Ar_), 128.0 (CH_Ar_), 126.6 (CH_Ethylenic_), 123.5 (CH_Ar_), 122.7 (CH_Ar_), 122.6 (CH_Ar_), 116.6 (Cq), 115.7 (CH_Ar_), 53.0 (CH_2_), 52.6 (CH_2_), 37.78 (CH_3_), 20.6 (CH_3_). **MS:** 388.03659 [M + 1]+; **FT-IR (υ in cm^−1^):** 2964 (Aliphatic C–H), 1720 (C=O Ester), 1660 (C=O Ketone in quinolone), 1447 (Aromatic C=C stretching), 3136 (Aromatic C-H stretching), 1244 (C-N triazole bond); UV–vis: λ_max_ = 362 nm.
*Methyl 2-Oxo-1-((1-(4-(trifluoromethyl)benzyl)-1H-1,2,3-triazol-4-yl)methyl)-1,2-dihydroquinoline-4-carboxylate* (**4c**). **Yield (%) =** 85%; **mp:** 503–505 K; **NMR ^1^H (300 MHz, CDCl_3_): δ** (ppm) 8.1 (s, 1H, CH_Ar_), 8.9 (dd, *J* = 8.1, 1.5 Hz, 1H, CH_Ar_), 7.8–7.6 (m, 5H, CH_Ar_), 7.4 (d, *J* = 8.0 Hz, 2H, CH_Ar_), 7.3–7.3 (m, 1H, CH_Ar_), 7.0 (s, 1H, CH_Ethylenic_), 5.6 (s, 2H, CH_2_), 5.5 (s, 2H, CH_2_), 3.9 (s, 3H, CH_3_). **^13^C NMR APT (75 MHz, CDCl_3_): δ** (ppm) 165.3 (C=O_ester_), 159.9 (C=O_Amide_), 142.7 (Cq), 140.5 (Cq), 139.7 (Cq), 139.2 (Cq), 131.4 (CH_Ar_), 128.6 (CH_Ar_), 127.8 (CH_Ethylenic_), 126.5 (CH_Ar_), 125.6 (CH_Ar_), 124.0 (CH_Ar_), 122.6 (CH_Ar_), 122.5 (CH_Ar_), 116.5 (CH_Ar_), 53.0 (CH_3_), 52.1 (CH_3_), 37.7 (CH_3_). **MS:** 441.01463 [M + 1]+; **FT-IR (υ in cm^−1^):** 2959 (Aliphatic C–H), 1730 (C=O Ester), 1642 (C=O Ketone in quinolone), 1436 (Aromatic C=C stretching), 3124 (Aromatic C-H stretching), 1323 (C-F stretching), 1242 (C-N triazole bond); **UV–vis**: λ_max_ = 327 nm.

### 2.5. Antibacterial Activity

#### 2.5.1. Disc Diffusion Method

In this method, Sterile Whatman No. 1 disks (6 mm diameter) were carefully placed on the agar surface after the medium had rested for 15 min. Each disc was then loaded with 10 µL of the novel synthesized compounds **4a**–**4c** (10 mg/mL in DMSO). The Petri dishes were then incubated at 37 °C for 24 h. The detection of a translucent halo around the disc, indicating inhibited bacterial growth, was used to measure antimicrobial activity after the incubation period. A caliper was used to measure the diameter of the halo in millimeters [42].

#### 2.5.2. Minimum Inhibitory Concentration

The minimum inhibitory concentrations (MIC) of the newly synthesized compounds were determined using a modified method based on the approach by Bouhdid et al. [43] Serial dilutions of the compounds were prepared in 100% pure DMSO, ensuring that the final concentration of DMSO in each well did not exceed 1% (*v*/*v*) to prevent any potential interference with bacterial growth. Each well of a sterile 96-well microplate contained 140 µL of Brain Heart Infusion (BHI) medium, 20 µL of a standardized bacterial inoculum, and an appropriate volume of the compound solution prepared in DMSO. This volume was adjusted to maintain the final DMSO concentration at ≤1% (*v*/*v*) in the total volume of each well. After incubating the plates at 37 °C for 24 h, bacterial growth was assessed visually based on turbidity. The MIC was defined as the lowest concentration of the compound at which no visible turbidity was observed, indicating complete inhibition of bacterial growth.

### 2.6. Drug-likeness and ADMET Prediction

Predicting ADMET properties is a critical part of computational drug discovery. This process provides the opportunity to obtain necessary information about the biological efficacy and safety of potential drug candidates, thereby reducing both the time and cost of experimental research [44,45]. By evaluating these parameters, researchers can clarify the likelihood of new drugs with greater confidence.

As part of this work, the SwissADME server and the pkCSM web resource were utilized to evaluate the physicochemical and pharmacokinetic properties of the investigated compounds, thereby predicting their efficacy and potential as viable drug candidates.

### 2.7. Molecular Docking Studies

The molecules were designed using ChemSketch, followed by geometry optimization using the MM2 force field [46]. These compounds were then tested for antibacterial activity. The LasR-OC12 HSL receptor, obtained from PDB ID: 3IX3 (https://doi.org/10.2210/pdb3IX3/pdb), was used in the antibacterial study. A lattice with dimensions of X = 25.5149 Å, Y = 2.8599 Å and Z = 12.7483 Å was defined for the docking analyses. Molecular docking was conducted using AutoDock tools with a grid size of 63 × 47 × 60 along the x, y, and z axes [47]. Before docking, the molecules and their receptor complexes were carefully prepared, including the removal of water molecules.

The docking protocol simulated potential ligand–protein interactions, and the analysis was refined using Discovery Studio, version 2021 (BIOVIA, Dassault Systèmes, San Diego, CA, USA) [41]. This refinement included removing residual water molecules, addressing incomplete side chain residues, and improving structural accuracy by adding non-polar hydrogens. These improvements increased the accuracy and reliability of the results.

### 2.8. Molecular Dynamics Simulations

In this study, molecular dynamics simulations (MDS) were conducted using the Desmond software package, version 2021.3 (Schrödinger, LLC, New York, NY, USA), which is part of the academic version of the Schrödinger Suite. The initial step involved energy minimization of the ligand-receptor complexes using the OPLS3e force field [48]. The systems were then solvated in an orthorhombic water box, ensuring a minimum distance of 10 Å between the solute and the box boundaries. Water molecules were represented using the SPC solvent model. Sodium (Na^+^) and chloride (Cl^−^) ions were added to balance the overall charge of the system and to adjust the salt concentration to 1.15 M, simulating physiological conditions. A brief additional minimization followed this [49]. Subsequent simulations were carried out in an NPT ensemble using the Nosé–Hoover thermostat. The temperature was gradually raised to 300 K while maintaining a pressure of 1.013 bar. Molecular dynamics simulations were run for 100 ns, with trajectory frames stored at 10 ps intervals and energy data recorded every 1.2 ps [50,51,52]. Utilizing Desmond’s “Simulation Interaction Diagram,” the study thoroughly examined protein–ligand interactions throughout the simulation, providing a deeper understanding of key binding interactions.

## 3. Results and Discussion

### 3.1. QSAR Modelling

Using the MLR model, only one descriptor was chosen out of 123 to best describe the variability in the IC_50_ independent variable: FractionCsP^3^ (Table 1). The fractionCsP^3^ is a descriptor used in medicinal chemistry to assess the saturation level of a molecule. It is defined as the ratio of sp^3^-hybridized carbon atoms to the total number of carbon atoms in the structure. In this study, which focuses on the design of new 2-quinolone derivatives, reducing the CsP3 fraction is crucial for achieving lower IC_50_ values and thereby enhancing antibacterial activity against various bacterial strains. A lower CsP^3^ fraction may contribute to increased molecular rigidity, improved target binding through π-π interactions or hydrogen bonding, and better alignment with the structural features of highly active quinolones.

The two-sided Pearson correlation test was used to determine whether a correlation existed between the FractionCSP3 and the IC_50_ variables, and the results revealed a Pearson product-moment correlation coefficient of 0.69. To assess the significance of the correlation, a Student’s *t*-test was used, yielding a *p*-value of 2.87 × 10^−5^, indicating that the correlation is statistically significant. After determining the significance of the correlation between both variables, a symbolic regression model was created, resulting in the following mathematical equation:

**IC_50_** = 8.41 × exp (2 × FractionCSP3) − 8.94 (1)

The R^2^ and RMSE metrics from training, cross-validation, and testing (Table 2) were calculated to assess the quality of the equation. The R^2^ value in both training and prediction exceeds the threshold of 0.61. The RMSE values are low and acceptable for both training and prediction.

**Figure 1:**
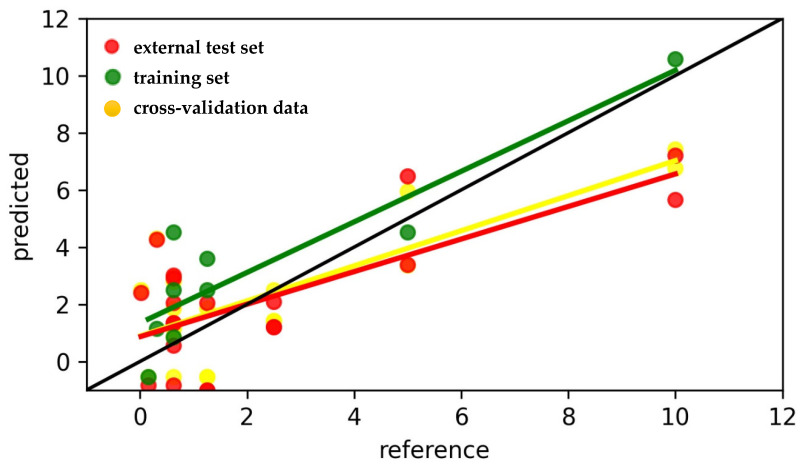
Reference vs. predicted values.

After evaluating the performance metrics of the developed QSAR model, Figure 1 was used to analyze further the distribution of predicted versus experimental IC_50_ values across the full range of compounds. The figure highlights the model’s predictive behavior, particularly for compounds exhibiting high IC_50_ values. Specifically, compounds with IC_50_ values greater than 8 were systematically underestimated during both the training and cross-validation phases. This underestimation suggests a potential limitation in the model’s ability to accurately learn from or generalize high IC_50_ data within these datasets. Interestingly, this issue was not observed in the prediction (external test) phase, where all compounds, including those with high IC_50_ values, were accurately predicted. This improved predictive accuracy on the test set may reflect the absence of experimental noise or data uncertainty in those specific compounds compared to those in the training and validation sets. Indeed, the discrepancy could stem from considerable experimental variability or measurement errors associated with IC_50_ values in the training and cross-validation datasets, which may have affected the model’s learning process. Despite this observed trend, the model’s overall performance remains robust. The correlation between predicted and reference values, as shown in Figure 1, supports the reliability of the model. The consistent performance across training, validation, and test sets suggests the model is not overfitting and is capable of generalizing to unseen data. Thus, these findings reinforce the model’s validity and demonstrate its readiness for practical application in predicting the IC_50_ values of new, untested compounds.

### 3.2. Synthesis of Novel 2-Oxo-1,2-dihydroquinoline Derivatives

**Scheme 3:**
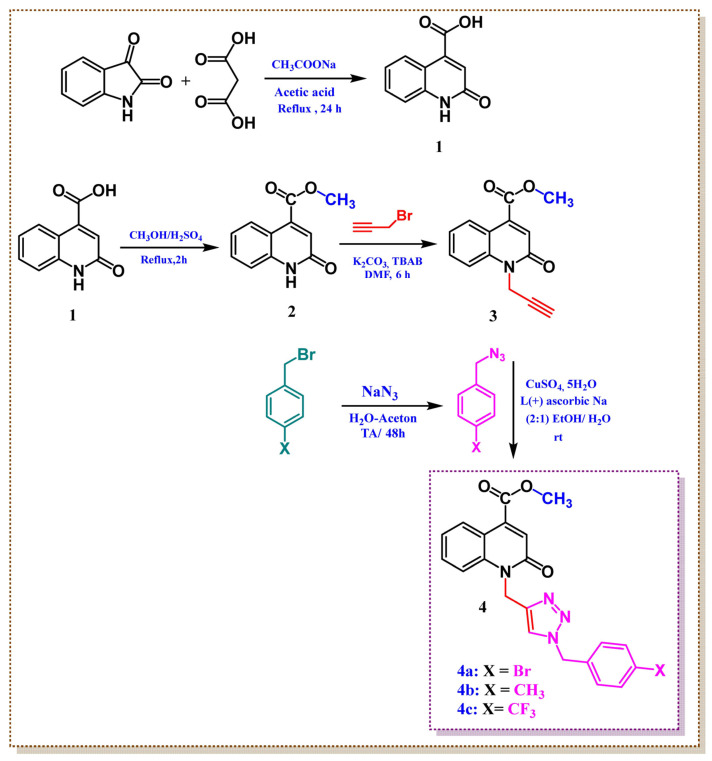
Synthesis of new methyl 1-((1x-benzyl-1H-1,2,3-triazol-4-yl)methyl)-2-oxo-1,2-dihydroquinoline-4-carboxylate derivatives (**4a**–**4c**).

Guided by our established QSAR model, we have selected to synthesize a novel series of compounds that incorporate quinolone and triazole frameworks. The synthesis of the new methyl 1-((1x-benzyl-1H-1,2,3-triazol-4-yl)methyl)-2-oxo-1,2-dihydroquinoline-4-carboxylate derivatives (**4a**–**4c**) was executed in four stages, as illustrated in Scheme 3. The two intermediates, esterification 2 and alkylation 3, were synthesized following the reported procedure [41]. The synthetic route commenced with the formation of 2-oxo-1,2-dihydroquinoline-4-carboxylic acid (compound **1**), which was achieved by condensing malonic acid with isatin in acetic acid under reflux, using sodium acetate as a base. After isolating and purifying the acid product, esterification was performed by treating it with methanol and a few drops of concentrated sulfuric acid, yielding compound **2**. This intermediate was then subjected to N-alkylation under phase-transfer conditions using propargyl bromide as the alkylating agent, in the presence of TBAB, potassium carbonate, and DMF at room temperature for 6 h, furnishing the propargylated product **3** in good yield. The synthesis of novel methyl 1-((1x-benzyl-1H-1,2,3-triazol-4-yl)methyl)-2-oxo-1,2-dihydroquinoline-4-carboxylate derivatives (**4a**–**4c**) via 1,3-dipolar cycloaddition has been accomplished. The reaction of these derivatives with several alkyl azides (4-bromobenzyl azide, 4-methylbenzyl azide, and trifluoromethylbenzyl azide), which were synthesized beforehand, with the alkylated product **3**, was conducted utilizing eco-friendly methods in a 1:1 ethanol/water mixture and a cost-effective catalyst, CuSO_4_·5H_2_O, at ambient temperature.

Thin-layer chromatography (TLC) was used to monitor the progress of the reactions. The synthesized compounds underwent purification via liquid chromatography on silica gel, utilizing the gel as the stationary phase. The various chemical structures were elucidated using standard spectroscopic methods.

### 3.3. UV-Vis Spectrum

**Figure 2:**
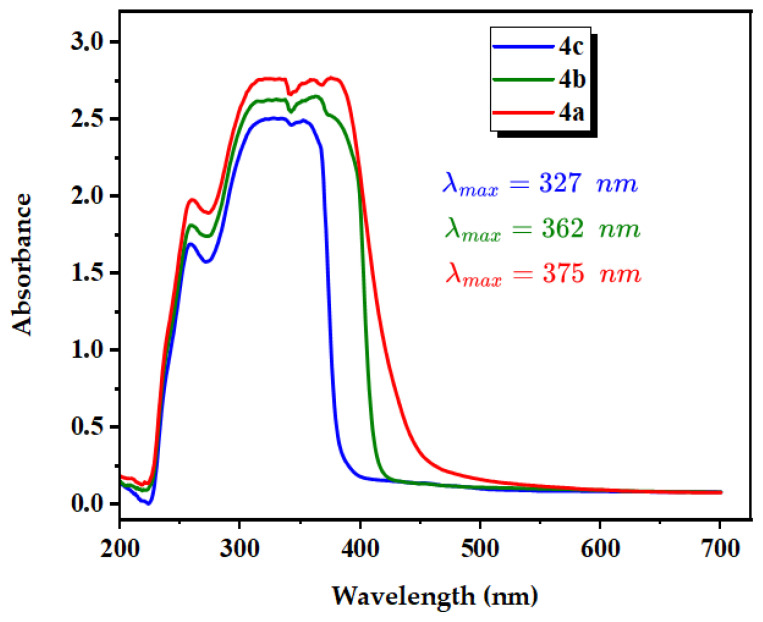
UV-Vis spectrum of compounds **4a**–**4c**.

UV-Vis analysis of the three synthesized compounds (**4a**–**4c**) revealed maximum absorption wavelengths (λ_max_) at 375, 362 and 327 nm, respectively. The bathochromic shift observed from **4c** to **4a** indicates an increase in electron conjugation, as well as the presence of electron-donating groups by mesomeric effects (Br and CF_3_), which enhances π-electron delocalization. This delocalization decreases the HOMO-LUMO energy difference, resulting in absorption at longer wavelengths. These results indicate that the electronic properties of the substituents have a significant influence on the optical properties of the synthesized compounds, particularly on their absorption behavior in the UV region (Figure 2).

### 3.4. Drug-likeness and ADMET Results

Many drug development failures are attributed to poor pharmacokinetics and bioavailability, in addition to problems with efficacy and toxicity [53,54]. Among the key pharmacokinetic properties to be evaluated, human intestinal absorption (HIA) and the ability to cross the blood–brain barrier (BBB) are particularly critical. To predict these properties, the Egan boiled egg method has been proposed. The model is based on an analysis of the lipophilicity and polarity of small organic molecules [55]. The technique can be applied at various stages of drug discovery, from screening chemical libraries to evaluating drug candidates in the development phase.

**Figure 3:**
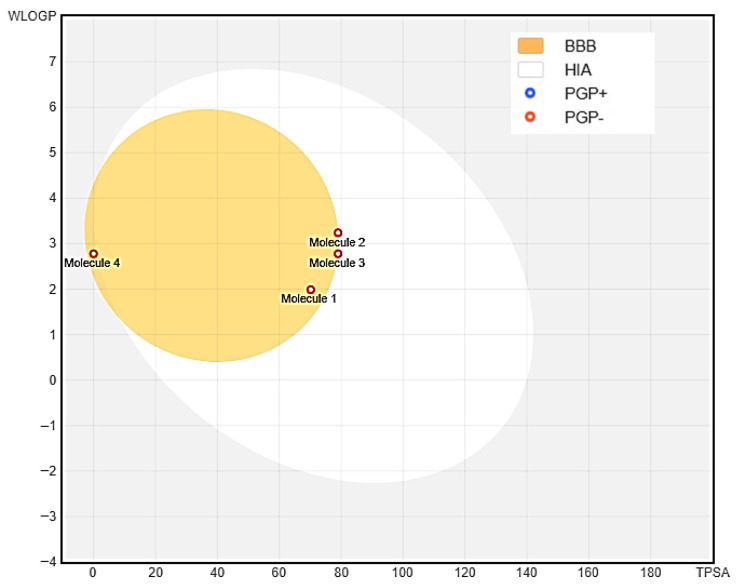
Predictive model of Egan Boiled Egg for the ligands (molecule **1**: **Q3**, molecule **2**: **4a**, molecule **3**: **4b** and molecule **4**: **4c**).

The predictive model of the Egan boiled egg shown in Figure 3 indicates that three ligands (**Q3**, **4a**, **4b,** and **4c**) are located within the yolk portion of the model. This positioning suggests that the ligands have a strong ability to cross the blood–brain barrier (BBB) and can also be effectively cleared from the central nervous system (CNS) via P-glycoprotein activity. This finding supports its potential as a viable pharmaceutical candidate, as efficient intestinal absorption is crucial for systemic drug distribution and overall therapeutic efficacy.

Additionally, the Drug Bioavailability Radar Mapping tool is utilized in the field of drug development and discovery to evaluate the bioavailability and drug-like properties of a compound. This visual tool provides a graphical representation of crucial physicochemical properties, enabling researchers to determine whether a molecule falls within the optimal range for oral bioavailability. The Radar is based on six parameters: lipophilicity (LIPO) for hydrophobicity assessment, Size (SIZE) for molecular weight determination, polarity (POLAR) for polarity analysis, insolubility (INSOLU) for aqueous solubility prediction, unsaturation (INSATU) for saturation level assessment, and flexibility (FLEX) for structural flexibility assessment based on the number of rotatable bonds [56,57,58].

**Figure 4:**
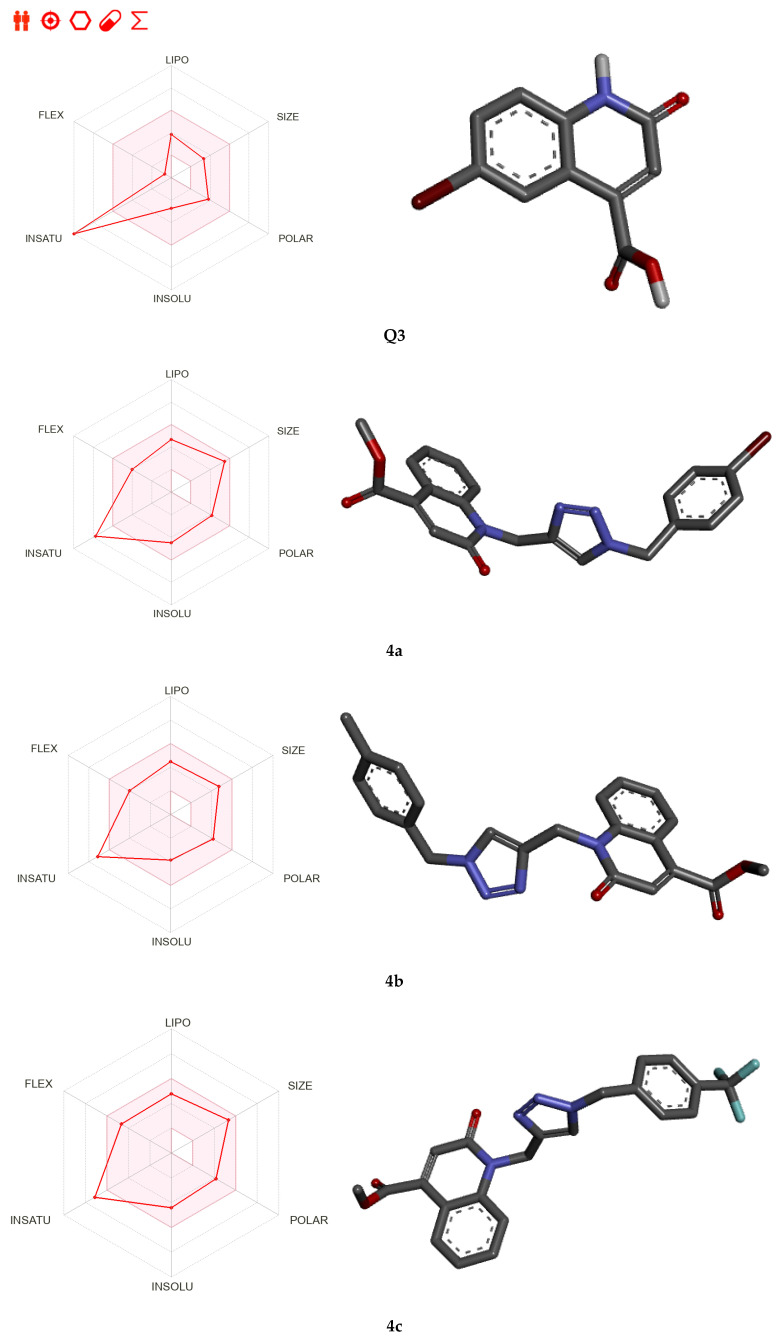
Bioavailability radar graphs and the stable structure of the four ligands (**Q3**, **4a**, **4b** and **4c**).

Examining the bioavailability radars shown in Figure 4 for the analyzed and synthesized molecules (**Q3**, **4a**, **4b**, and **4c**), we observe that the synthesized ligands **4a**, **4b**, and **4c** exhibit good oral bioavailability, with the ligand falling within the pink area. We can consider these molecules as promising candidates for the synthesis of antibiotics. Molecule **Q3** is close to being considered a drug candidate, but there is a slight deviation from the white zone at the point of unsaturation. By adding new pharmacophores to this compound, the graphical line can be drawn entirely in the pink zone.

Ten distinct parameters, ranging from molecular weight to molar refractivity, were analyzed by Lipinski’s rule of five. The molecular weights of these four ligands range from 203 to 443 g/mol, as shown in Table 3. Compound **Q3** exhibits a lower number of heavy atoms, with only 15 atoms, while molecule **4c** has the highest number of heavy atoms, with 32. The number of heavy aromatic atoms of molecules **4a**, **4b** and **4c** ranges from 10 to 21, with molecules **4a**, **4b**, and **4c** exhibiting a maximum of 21 atoms. The Csp^3^ fraction varies between 0 and 0.19, with molecule **Q3** having a value of 0. The number of rotating bonds falls within the range of 1 to 7 for all molecules. Furthermore, all molecules have fewer than 10 hydrogen bond acceptors/donors, whereas the synthesized molecules have no hydrogen bond donors. Molar refractivity values range from 59.30 to 79.01 Å^2^; the synthesized molecules exhibit the highest value of 79.01 Å^2^. The partition coefficient (Log P) value ranges from 1.2367 to 3.4950, which indicates strong lipophilic properties. This coefficient is used to evaluate how the drug distributes between two non-miscible phases, blood and tissues. Finally, the TPSA value ranges from 59.23 to 112.27 Å^2^ (Table 3). All four molecules showed relevant physicochemical properties, particularly the three synthesized molecules. The analysis of the physicochemical properties of the four molecules reveals adaptable characteristics with good oral bioavailability. The molecular weights and log *p* values indicate a balanced profile between solubility and membrane permeability. The moderately polar surface areas suggest a favorable ability to cross biological barriers. In addition, the low number of hydrogen bond acceptors and donors, coupled with a limited number of rotatable bonds, suggests sufficient molecular flexibility for stable and efficient binding to the target. In particular, the synthesized ligands exhibit high molar refractive index, reinforcing their potential for critical therapeutic applications. Consequently, the results obtained confirm that these new molecules open the door to promising new synthetic routes as drug candidates.

The four bioactive compounds (**Q3**, **4a**, **4b**, and **4c**) are presently being analyzed via in silico ADMET experiments utilizing pkCSM and ADMEswiss [59]. Their predicted ADMET properties are summarized in Table 4, highlighting numerous significant findings. All compounds demonstrate significant absorption rates (95–99%), suggesting substantial potential for human gastrointestinal absorption, especially compound **4b**. Also, they exhibit considerable water solubility, with **Q3** (−2.73 Log mol/L) demonstrating the greatest solubility.

The compounds show significant penetration in BBB permeability. However, with Log PS values ranging from −2.206 to −2.686, CNS permeability is moderate. The distribution of these molecules throughout the BBB and central nervous system is greatly influenced by their lipophilic and polar properties, which are influenced by halogens such as Br and CF3 and alkyl substituents.

Furthermore, CYP3A4, a crucial enzyme involved in drug metabolism, may be both a substrate and an inhibitor target of the compounds [60]. By altering elimination rates and therapeutic duration, this dual role may affect their pharmacokinetics. Most compounds have longer retention times according to clearance indices, except **4b** (0.749), which may increase their effectiveness at lower dosages.

Toxicity assessments, including the Ames test, hepatotoxicity, and skin sensitization, revealed no mutagenic or skin sensitization risks (Table 4). However, the synthesized compounds (**4a**, **4b,** and **4c**) showed hepatotoxicity. Despite this, all compounds met the evaluated pharmacokinetic criteria, highlighting their potential as drug candidates. These findings will guide further optimization and development for therapeutic applications.

### 3.5. Molecular Docking Results

**Figure 5:**
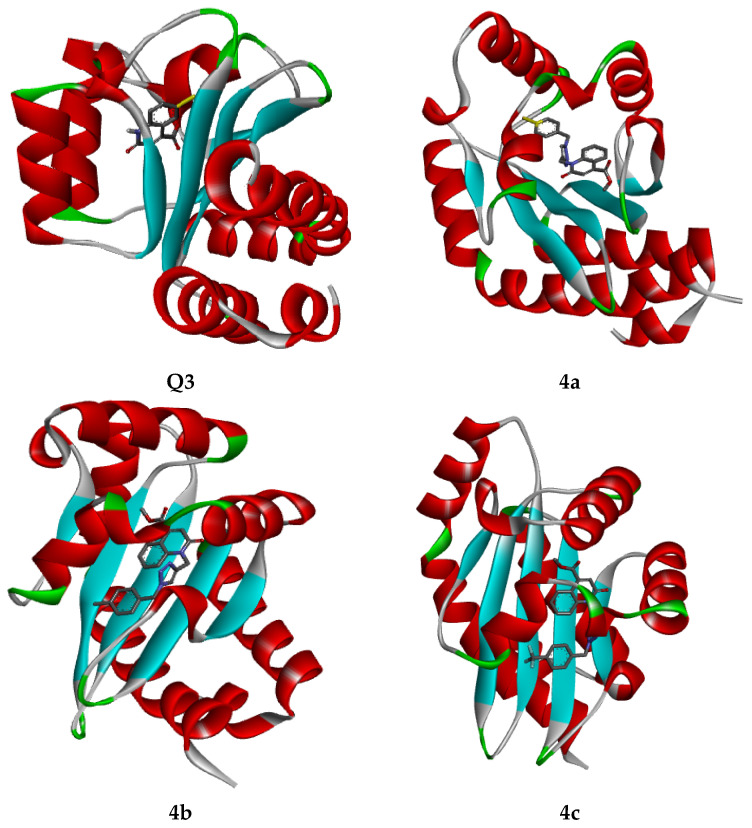
Three-dimensional representation of active compound binding to LasR-OC12 (PDB: 3IX3).

**Figure 6:**
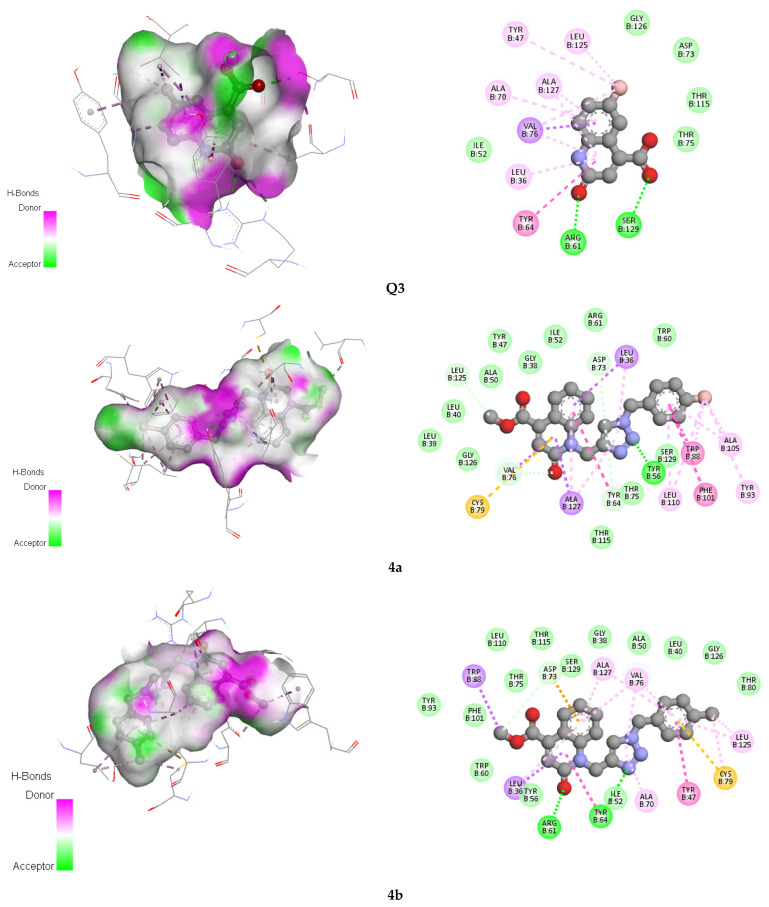

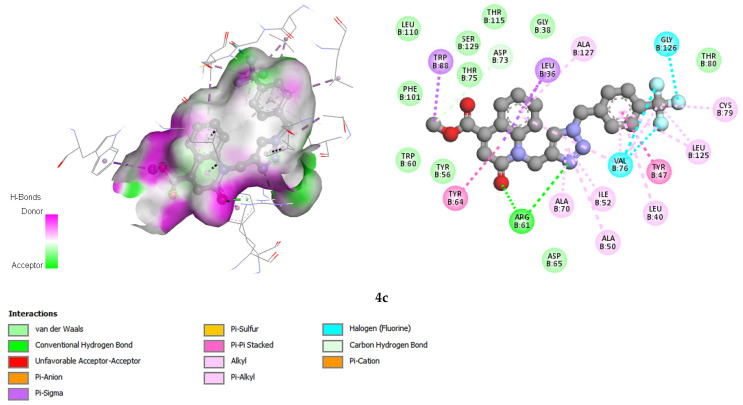
Two-dimensional and 3D representations of the resulting interactions between the active compounds (**Q3**, **4a**, **4b**, and **4c**) upon complexation with the LasR-OC12 HSL receptor (PDB ID: 3IX3).

A molecular docking study was conducted to validate further the results of the QSAR analysis regarding the significant antibacterial activity of the selected compounds, **Q3**, and its synthesized derivatives (**4a**, **4b,** and **4c**). In this study, the protein chosen as a potential target for the quinolone derivatives included the HSL receptor LasR-OC12 (PDB ID: 3IX3). The docking results, including 2D and 3D visualizations, are clearly presented in Figure 5 and further summarized in Figure 6 and Table 5.

The significant results of this study highlight the different interactions between the ligands (**Q3**, **4a**, **4b,** and **4c**) and their target protein (LasR-OC12), revealing significant differences in binding energy and types of molecular interactions. Among the synthesized ligands, **4a**, **4b,** and **4c** stand out for their exceptional binding affinity (−9.4 Kcal/mol for **4a**, −9.2 Kcal/mol for **4b**, and −8.2 Kcal/mol for **4c**), indicating a powerful interaction with the LasR-OC12 protein. This significant affinity can be attributed to the nature of the interactions established, including various conventional hydrogen bonds with target residues such as Tyr B56 with distances of 2.34 Å (**4a**), Tyr B64 with a distance of 2.78 Å, Arg B61 with distances of 2.12 Å (**4b**), and Arg B61 (**4c**) with distances of 2.07 Å, respectively, as well as hydrophobic contacts such as pi-sigma interactions with: Leu B36 with a distance of 3.93 Å, Ala B127 with a distance of 3.82 Å (**4a**), Leu B36 with a distance of 3.83 Å, Trp B88 with a distance of 3.91 Å (**4b**), Leu B36 at a distance of 3.97 Å, Trp B88 at a distance of 3. 82 Å (**4c**), and other key interactions such as alkyl and pi-alkyl interactions with Leu B110, Tyr B93, Ala B105, Val B76, Ala B127, Ala B70, Val B70, Ile B52, Leu B40, Ala B50, and Cys B79. In addition, specific interactions such as Pi-Sulfur (Cys B79), Pi-Anion (Asp B73), and Halogen (Gly B126 and Val B76). These different interactions play a key role in stabilizing the ligand-receptor complex. In contrast, the ligand **Q3** has the lowest binding affinity, with a value of −7.9 kcal/mol, likely due to the fewer stabilizing interactions. However, it should be noted that all ligands, including **Q3**, interact with key protein residues, such as conventional hydrogen bonds with Ser B129 and Arg B61 at distances of 3.04 Å and 2.22 Å, respectively; Pi-Sigma with Val B76 at distances of 3.73 Å; and other interactions with various residues such as Ile B52, Thr B75, Thr B115, Asp B73, and Gly B126. Highlighting the importance of these residues in molecular recognition. Overall, these findings provide valuable insight into how ligands bind to the target protein. The observed variations in binding strengths and interaction types offer a solid foundation for designing more effective compounds. By focusing on critical residues and optimizing hydrophobic and electrostatic interactions, these results could be instrumental in developing new therapeutic molecules that precisely modulate the protein’s activity.

### 3.6. Antibacterial Activity (Results and Discussion)

**Figure 7:**
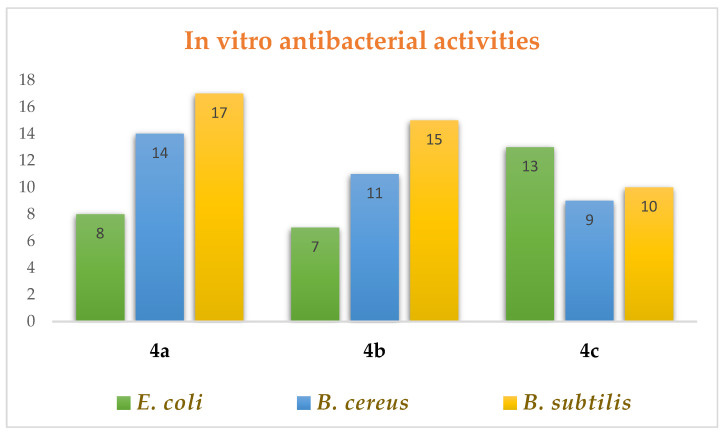
In vitro antibacterial activities of compounds **4a**–**4c** against *E. coli* (ATCC 25922), *B. cereus* (ATCC 9634) and *B. subtilis* (ATCC 3366), expressed as inhibition zone diameters (mm). Each value corresponds to the mean diameter of the clear zone around the disc. Results indicate that compound 4a exhibited the largest inhibition zones, particularly against *B. subtilis* (17 mm).

The methyl 1-((1-benzyl-1H-1,2,3-triazol-4-yl)methyl)-2-oxo-1,2-dihydroquinoline-4-carboxylate derivatives (**4a**–**4c**) were evaluated for their in vitro antibacterial activity against both Gram-positive and Gram-negative bacteria. The minimum inhibitory concentrations (MICs) were determined and are presented in Table 6 and Figure 7.

All the synthesized compounds exhibited antibacterial activity against *Escherichia coli* (ATCC 25922), *Bacillus cereus* (ATCC 9634), and *Bacillus subtilis* (ATCC 3366). Among them, compound **4a** showed the highest activity, with MIC values of 19 µg/mL against *B. subtilis*, 38 µg/mL against *B. cereus*, and 155 µg/mL against *E. coli*. Compound **4b** followed, with MICs of 38 µg/mL, 75 µg/mL, and 315 µg/mL against the same respective strains. Compound **4c** exhibited lower activity, with MICs of 315 µg/mL for *B. subtilis* and *B. cereus*, and 1250 µg/mL for *E. coli*.

For reference, the MIC of ciprofloxacin, a standard antibiotic used as a control, was 12–15 µg/mL across the tested bacterial strains. It is essential to note that ciprofloxacin remains significantly more potent than our compounds, as it exhibits inhibitory effects at substantially lower concentrations. This comparison was clarified to avoid any misinterpretation due to unit inconsistencies in the previous version.

The observed antibacterial differences among compounds **4a**–**4c** can be attributed to structural variations. These derivatives result from the conjugation of two pharmacologically relevant scaffolds: 2-quinolone, a core structure in many antibiotics, and 1,2,3-triazole, known for its bioactive properties. Additionally, the nature of the substituents at the para-position of the benzyl group plays a key role in modulating activity.

Compound **4b**, which carries a methyl group, likely benefits from enhanced hydrophobic interactions with the LasR-OC12 HSL receptor, improving its antibacterial effect. In contrast, compounds **4a** and **4c** bear electron-withdrawing groups (Br and CF_3_, respectively), which may contribute to binding affinity and biological activity.

These in vitro results are consistent with the molecular docking findings, which revealed that compound **4a** had the most stable interaction with the target receptor (binding energy: −9.4 kcal/mol), compared to compound **4b** (−9.2 kcal/mol) and **4c** (−8.2 kcal/mol). In conclusion, although less potent than ciprofloxacin, these novel hybrid molecules show promise as lead structures for the development of next-generation antibacterial agents. Their unique scaffolds may help circumvent bacterial resistance mechanisms, including target site mutations and efflux pump activity. Further pharmacological investigations and structural optimization are warranted to assess their therapeutic potential fully.

### 3.7. Molecular Dynamics Simulation

In aqueous environments, molecular dynamics simulation (MDSs) is a potent computational method for assessing the dynamic stability and behavior of ligand–receptor complexes. In this work, an MDS was performed to investigate the stability and interaction patterns of compounds **4a**, **4b**, and **4c** inside the active site of the *Escherichia coli* protein (PDB ID: 3IX3) across a 100-nanosecond simulated period. To closely reflect physiological conditions and evaluate the conformational flexibility and binding stability of the ligand–protein complexes throughout the entire trajectory, the simulations were carried out in an explicit solvent environment.

**Figure 8:**
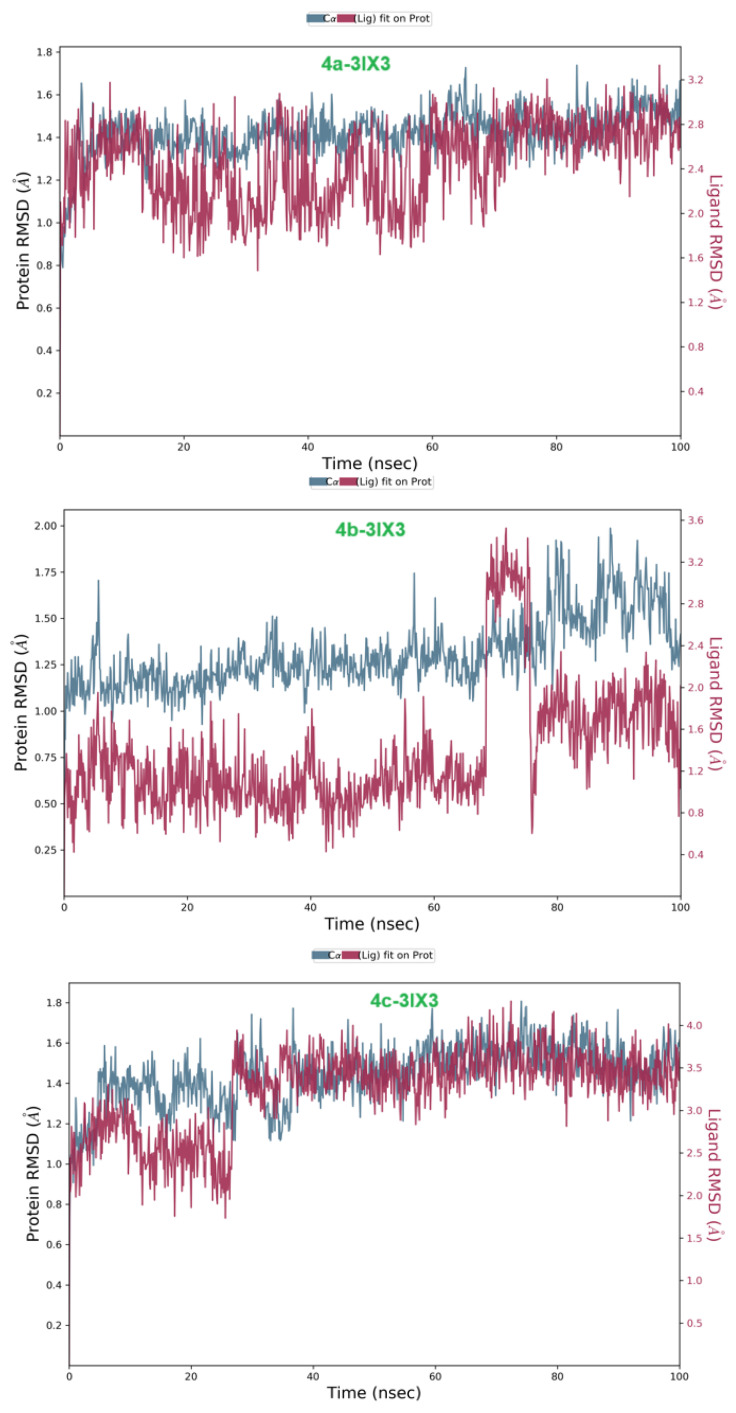
Root mean square deviation (RMSD) of the **4a-3IX3**, **4b-3IX3**, and **4c-3IX3** complexes over 100 ns.

From Figure 8, we observe the variation in RMSD values for proteins with a resolution below 2 Å. These variations are low, indicating that the proteins in the **4a-3IX3**, **4b-3IX3**, and **4c-3IX3** complexes have undergone minimal division. We also note that the RMSD for the alpha carbon of the protein’s amino acid residues reaches equilibrium after 5 ns, indicating that the proteins do not change in conformation within the studied complexes and exhibit excellent stability. The RMSD of the 4a ligand in the **4a-3IX3** complex underwent small variations and reached its steady state throughout the simulation, indicating that ligand 4a exhibits excellent stability in the **4a-3IX3** complex. The RMSD of the **4b** ligand in the **4b-3IX3** varies between 0 Å and 3.6 Å for 100 ns and reaches its steady state between 0 ns and 70 ns, as well as between 80 ns and 100 ns. Indicating that ligand **4b** is stable over both time intervals. Between 70 ns and 80 ns, we observe a significant increase in the RMSD curve of the **4a** ligand, followed by a return to the steady state until the end of the simulation. This increase does not affect the ligand’s overall stability. The RMSD of ligand **4c** in the **4c-3IX3** complexes increases and then stabilizes after 25 ns, indicating that the ligand reaches its stable state after 25 ns.

**Figure 9:**
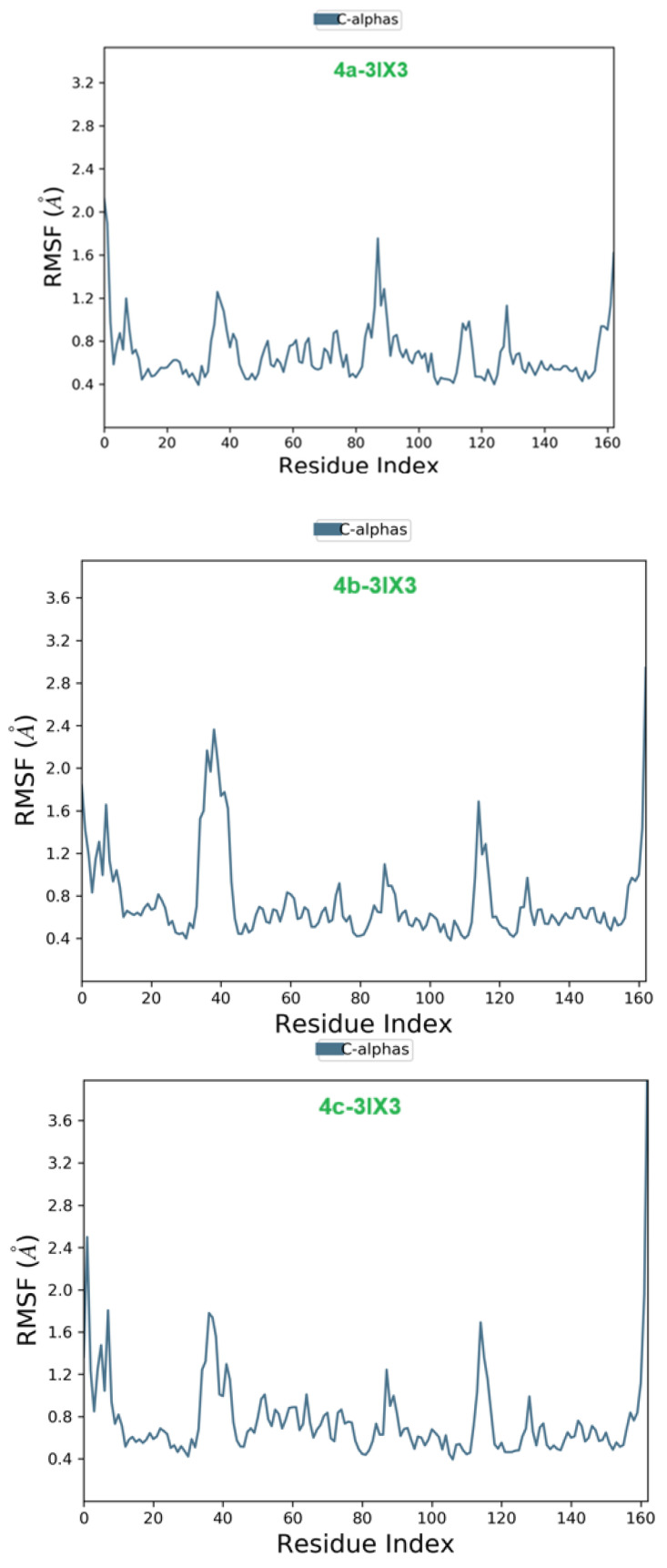
Root mean square fluctuation (RMSF) of the **4a-3IX3**, **4b-3IX3**, and **4c-3IX3** complexes over 100 ns.

From Figure 9, the RMSF plot of the alpha carbon of the protein’s amino acid residues in **4a-3IX3**, **4b-3IX3**, and **4c-3IX3** is almost similar. This figure shows that the majority of amino acid residues in the studied complexes have very low RMSF values, indicating that these residues are less fluctuating, confirming that complexes **4a-3IX3**, **4b-3IX3**, and **4c-3IX3** are stable. In this figure, we observe a few amino acid residues (residue indices between 1 and 10 and between 36 and 42, as well as between 156 and 162) with slightly elevated RMSD values, indicating that these residues exhibit slightly higher fluctuations, which their presence in flexible regions can explain. The mean RMSF values of complexes **4a-3IX3**, **4b-3IX3**, and **4c-3IX3** are 0.68 Å, 0.76 Å, and 0.77 Å, respectively. These mean values are very low, indicating that the high fluctuations observed at some amino acid residues do not significantly influence the overall stability of the complexes studied.

**Figure 10:**
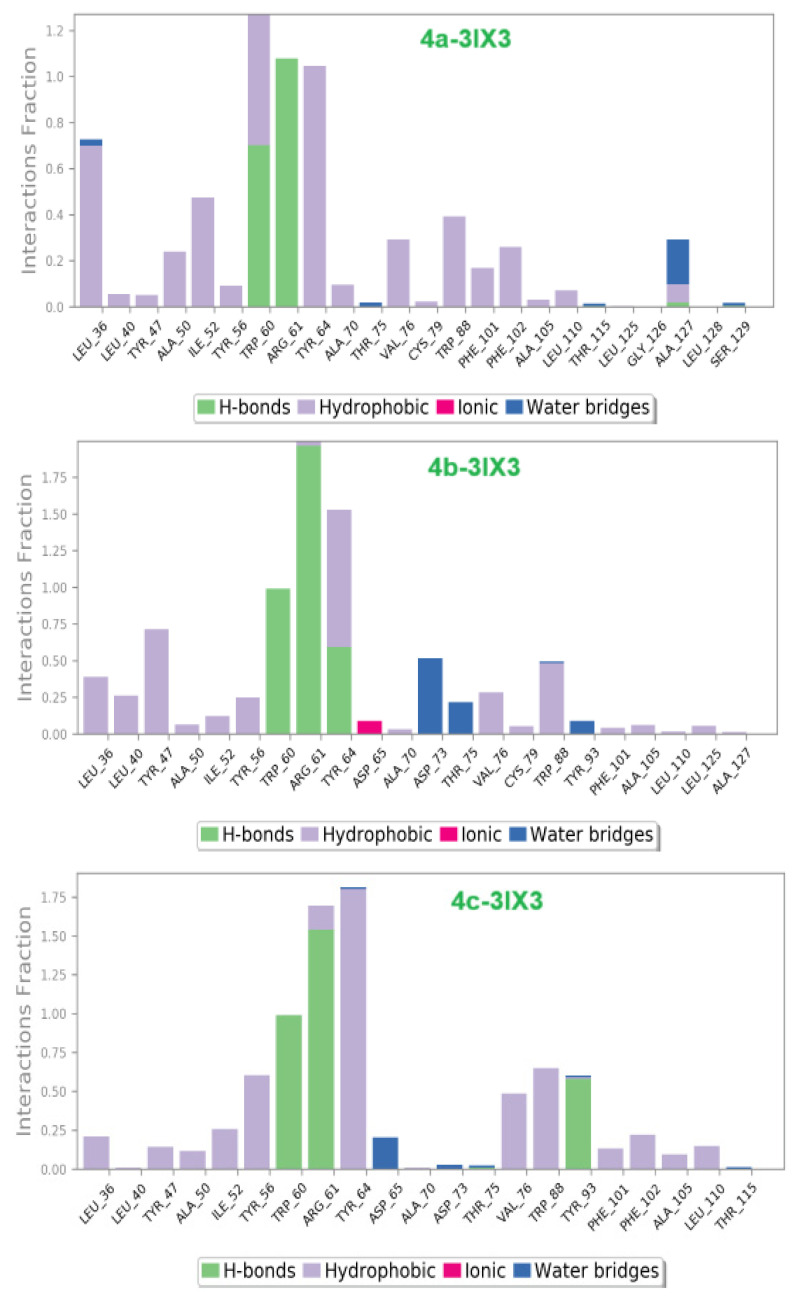
Histograms of interaction in the **4a-3IX3**, **4b-3IX3**, and **4c-3IX3** complexes over 100 ns.

From Figure 10, we observe that hydrogen and hydrophobic interactions contribute significantly. In contrast, water interactions contribute weakly to the stability of the **4a**, **4b,** and **4c** ligands in the active pocket of the *Escherichia coli* protein. These results show that hydrogen and hydrophobic interactions are more favorable to achieving high stability of the studied ligand in the active site of the *Escherichia coli* protein. From this figure, we also observe that the amino acid residues Trp60, Arg61, and Tyr64 present higher interaction fractions in the **4a-3IX3**, **4b-3IX3**, and **4c-3IX3** complexes, indicating that these residues play a significant role in the stability of ligands **4a**, **4b**, and **4c** in the active site of the *Escherichia coli* protein.

**Figure 11:**
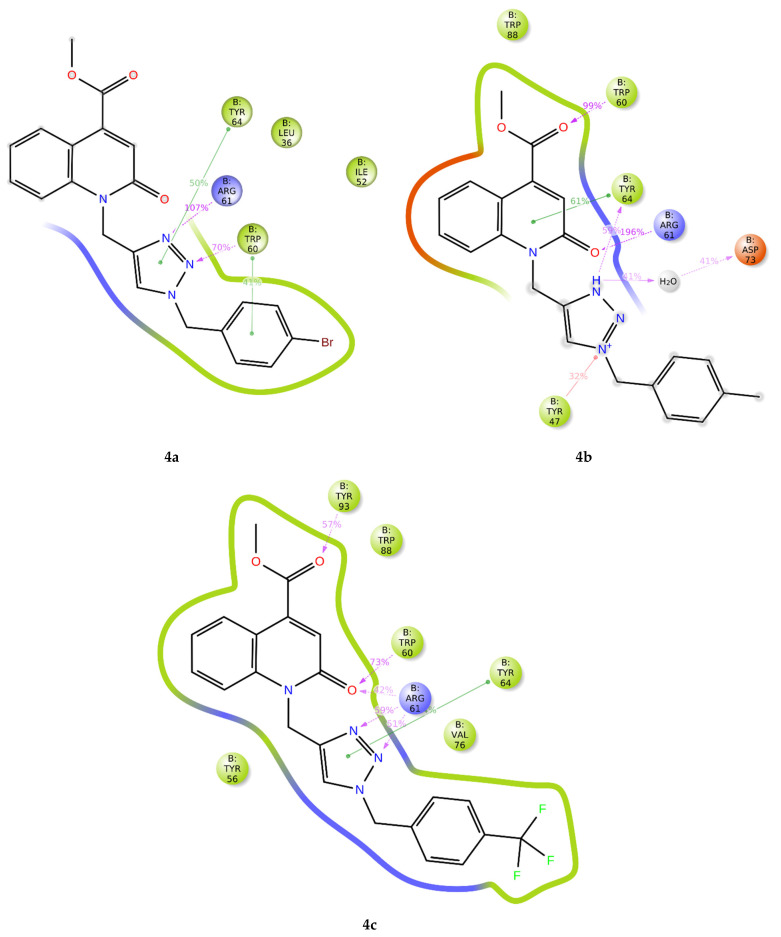
The 2D structure of formed complexes LasR-OC12 HSL-**4a**, LasR-OC12 HSL-**4b**, and LasR-OC12 HSL-**4c**.

Figure 11 illustrates the 2D binding interactions of the three synthesized compounds (**4a**, **4b** and **4c**) with the LasR-OC12-HSL receptor. All three compounds formed stable complexes thanks to multiple bonds, namely hydrogen bonds and hydrophobic contacts. Compound **4a** interacts mainly with residues such as Tyr, Leu and Ala. For example, compound **4b** exhibits a denser interaction network, including polar interactions and π-π stacking, which suggests a stronger binding affinity. And finally, Compound **4c** interacts in the same way through hydrogen bonding and hydrophobic forces, with additional stabilization due to its attracting group (CF3). The differences in bond types reflect the influence of substituents on molecular recognition. These results show that **4b** may have the highest binding strength, followed by **4c** and **4a**.

## 4. Conclusions

In this study, we successfully designed and synthesized a new series of quinolone–triazole (**4a**–**4c**) hybrid molecules, demonstrating their strong potential as novel antibacterial agents. Our integrated workflow, which included molecular docking, molecular dynamics simulations, and experimental validation, allowed us to predict and confirm their antibacterial activity along with favorable drug-like characteristics—compared to previously reported quinolone–triazole compounds, our molecules, especially compound **4a**, exhibited superior activity, with minimum inhibitory concentration values as low as 0.019 mg/mL against both Gram-positive and Gram-negative bacterial strains. These results, combined with excellent in silico ADMET profiles, underscore the therapeutic potential of our structural designs. Additionally, our QSAR-guided molecular design approach provided valuable insights into the relationship between structure and activity, which can be used to optimize this scaffold further. Future work will focus on conducting detailed cytotoxicity assays, target identification, and evaluation of in vivo efficacy to confirm the pharmacological potential of these compounds. We believe this study lays a strong foundation for the development of next-generation antibacterial agents based on the quinolone–triazole framework.

## Figures and Tables

**Table 1:** Suggested molecules.

Molecules	4a	4b	4c
Fraction CSP3	0.143	0.182	0.182

**Table 2:** Created QSAR models with their performance metrics.

Method	Model	Training		Testing	
		R^2^	RMSE	R^2^	RMSE
**SR**	IC_50_ = 8.41 × exp (2 × FractionCSP3) − 8.94 (1)	0.61	1.81	0.68	1.75

**Table 3:** Physicochemical and Drug-Likeness proprieties of the ligands (**Q3**, **4a**, **4b**, and **4c**).

Comp.	Formula	n. Heavy Atoms	n. Arom. Heavy Atoms	Fraction Csp3	n. Rotatable Bonds	Log P	n. H-Bond Acceptors	n. H-Bond Donors	TPSA	Molar Refractivity
**Q3**	C_10_H_6_BrNO_3_	15	10	0.00	1	1.9888	3	2	70.16 Å^2^	59.23
**4a**	C_21_H_17_BrN_4_O_3_	29	21	0.14	6	3.2387	5	0	79.01 Å^2^	112.27
**4b**	C_22_H_20_N_4_O_3_	29	21	0.18	6	2.7846	5	0	79.01 Å^2^	109.53
**4c**	C_22_H_17_F_3_N_4_O_3_	32	21	0.18	7	3.4950	8	0	79.01 Å^2^	109.57

**Table 4:** Prediction of the ADME-toxicity pharmacokinetic properties of the ligands (**Q3**, **4a**, **4b**, and **4c**).

Compounds/ADMET	Absorption		Distribution		Metabolism						Excretion	Toxicity		
					Cytochromes (CYP)									
					Substrate CYP		Inhibitor CYP							
	Water Solubility	Intestinal Absorption (Human)	BBB Permeability	CNS Permeability	2D6	3A4	1A2	3A4	2C9	2D6	Total Clearance	AMES Test of Toxicity	Hepatotoxicity	Skin Sensitization
Unity	(Log mol/L)	Numeric (% Abs)	Numeric (log BB)	Numeric (Log PS)	Categorical (Yes or No)						Log (mL/min/kg)	Categorical (Yes or No)		
**Q3**	−2.73	95.085	−0.385	−2.206	No	No	No	No	No	No	0.089	No	No	No
**4a**	−4.036	97.518	−1.077	−2.686	No	Yes	Yes	No	Yes	No	0.416	No	Yes	No
**4b**	−4.721	98.353	−1.315	−2.297	No	Yes	Yes	No	Yes	No	0.241	No	Yes	No
**4c**	−3.832	99.044	−0.893	−2.457	No	Yes	Yes	Yes	Yes	No	0.749	No	Yes	No

**Table 5:** Illustrates the different interactions established between ligands (**Q3**, **4a**, **4b,** and **4c**) and receptor residues (LasR-OC12 HSL).

Ligands	Molecular Formula	Binding Affinity (Kcal/mol)	Interaction Hydrogen-Binding	Hydrophobic Interaction
**Q3**	C_10_H_6_BrNO_3_	−7.9	Conventional H-bond: Ser B129, Arg B61	Pi-Sigma: Val B76 Alkyl and Pi-Alkyl: Leu B125, Tyr B47, Ala B127, Ala B70, Leu B36 Pi-Pi stacked: Tyr B64 Van der waals: Ile B52, Thr B75, Thr B115, Asp B73, Gly B126
**4a**	C_21_H_17_BrN_4_O_3_	−9.4	Conventional H-bond: Tyr B56 Carbon H-bond: Val B76, Tyr B76, Asp B63, Leu B125	Alkyl and Pi-Alkyl: Leu B110, Tyr B93, Ala B105 Pi-Pi stacked: Trp B88, Phe B101 Pi-Sigma: Leu B36, Ala B127 Pi-Sulfur: Cys B79 Van der waals: Leu B39, Leu B40, Gly B126, Ala B50, Gly B38, Ile B52, Arg B61, Trp B60, Ser B129, Thr B75, Thr B155 B120, Gly A120, Glu B124, Tyr B47, Ser B44, Lys B42
**4b**	C_22_H_20_N_4_O_3_	−9.2	Conventional H-bond: Tyr B64, Arg B61 Carbon H-bond: Val B76, Asp B73, Ala B127	Alkyl and Pi-Alkyl: Val B76, Ala B127, Ala B70 Pi-Pi stacked: Trp B70 Pi-Sigma: Leu B36, Trp B88 Pi-Sulfur: Cys B79 Pi-Anion: Asp B73 Van der waals: Leu B110, Leu B40, Gly B126, Ala B50, Gly B38, Ile B52, Trp B60, Ser B129, Thr B75, Thr B155 B120, Phe B101, Tyr B75
**4c**	C_22_H_17_F_3_N_4_O_3_	−8.2	Conventional H-bond: Arg B61 Carbon H-bond: Asp B73	Alkyl and Pi-Alkyl: Val B70, Ala B127, Ile B52, Leu B40, Ala B50, Cys B79 Pi-Pi stacked: Trp B47, Trp B64 Pi-Sigma: Leu B36, Trp B88 Halogen: Gly B126, Val B76 Van der waals: Leu B110, Ala B50, Gly B38, Trp B60, Ser B129, Thr B75, Thr B155, Phe B101, Tyr B75

**Table 6:** In vitro antibacterial activities of methyl 1-((1-benzyl-1H-1,2,3-triazol-4-yl)methyl)-2-oxo-1,2-dihydroquinoline-4-carboxylate derivatives (**4a**–**4c**) against *Escherichia coli* (ATCC 25922), *Bacillus cereus* (ATCC 9634), and *Bacillus subtilis* (ATCC 3366). Minimum inhibitory concentration (MIC) values are expressed in both µg/mL and µM. Ciprofloxacin was used as a reference antibiotic (MIC = 12–15 µg/mL ≈ 36–45 µM).

Compounds	*E. coli* ATCC 25922 (ZI)		*B. cereus* ATCC 9634 (ZI)		*B. subtilis* ATCC 3366 (ZI)	
	MIC (µg/mL)	MIC (µM)	MIC (µg/mL)	MIC (µM)	MIC (µg/mL)	MIC (µM)
**4a**	155	342	38	83	19	42
**4b**	315	811	75	193	38	98
**4c**	1250	2825	315	712	315	712
**Ciprofloxacin**	15	45	12	36	12	36

## Data Availability

The datasets used and/or analyzed during the current study are available from the corresponding author on reasonable request.

## Abbreviation

**QSAR**	Quantitative Structure–Activity Relationship
**ADMET**	Absorption, Distribution, Metabolism, Excretion and Toxicity
**MIC**	Minimum Inhibitory Concentration
**FT-IR**	Fourier Transform Infrared Spectroscopy
**UV-Vis**	Ultraviolet–Visible Spectroscopy
**MDS**	Molecular Dynamics Simulation
**SAR**	Structure–Activity Relationship
**NMR**	Nuclear Magnetic Resonance
**APT**	Attached Proton Test
**DMF**	Dimethylformamide
**RT**	Room Temperature
**TLC**	Thin Layer Chromatography
**TMS**	Tetramethylsilane
**BBB**	Blood–brain barrier
**HIA**	Human intestinal absorption
**CNS**	Central nervous system
**PDB**	Protein Data Bank
**TBAB**	Tetrabutylammonium bromide
